# Interference in multi-user optical wireless communications systems

**DOI:** 10.1098/rsta.2019.0190

**Published:** 2020-03-02

**Authors:** Iman Abdalla, Michael B. Rahaim, Thomas D. C. Little

**Affiliations:** 1 Department of Electrical and Computer Engineering, Boston University, Boston, MA, USA; 2 Department of Engineering, University of Massachusetts at Boston, Boston, MA, USA

**Keywords:** optical wireless communications, LiFi, interference, visible light communications

## Abstract

Visible light communications (VLC) (including LiFi) represent a subset of the broader field of optical wireless communications. Where narrow beams, typical of free space optical communications are largely free from interference. VLC encompasses use cases involving combined illumination and data access and supporting a wireless access point (AP) model. The use of many units provides scaling of spatial coverage for both lighting and data access. However, AP replication in close proximity creates many interference challenges that motivate the investigation embodied in this paper. In particular, we frame the interference challenge in the context of existing strategies for driving improvements in link performance and consider the impacts of multiple users, multiple sources and multiple cells. Lastly, we review the state of existing research in this area and recommend areas for further study.

This article is part of the theme issue ‘Optical wireless communication’.

## 1. Introduction

Optical wireless communications (OWC) have proven to be a robust technique for spanning primarily point-to-point links for such applications as building-to-building (fixed), vehicle-to-vehicle (mobile) or mixed endpoint communications. These are typically served by narrow beams that are more easily isolated to single target receivers. The introduction of divergent sources permits the possibility to serve multiple receivers with a single signal but also encourages interference in the presence of multiple sources. The emergence of light-emitting diode (LED) lighting as a basis for OWC for multiple users exacerbates the need to understand the impact of each of these dimensions: source divergence and the existence of neighbours. Research in this area spans visible light communications (VLC) [1,2] and light fidelity (LiFi) [3–5] and has led us to the point where functional OWC multiple access systems are now available for commercial deployment [6–8]. As an emerging technology, the research community has not fully explored the impact of large-scale VLC installations nor the impact of increasing densities of access points (APs) to support the growing number of devices desiring network access.

**Figure 1.**
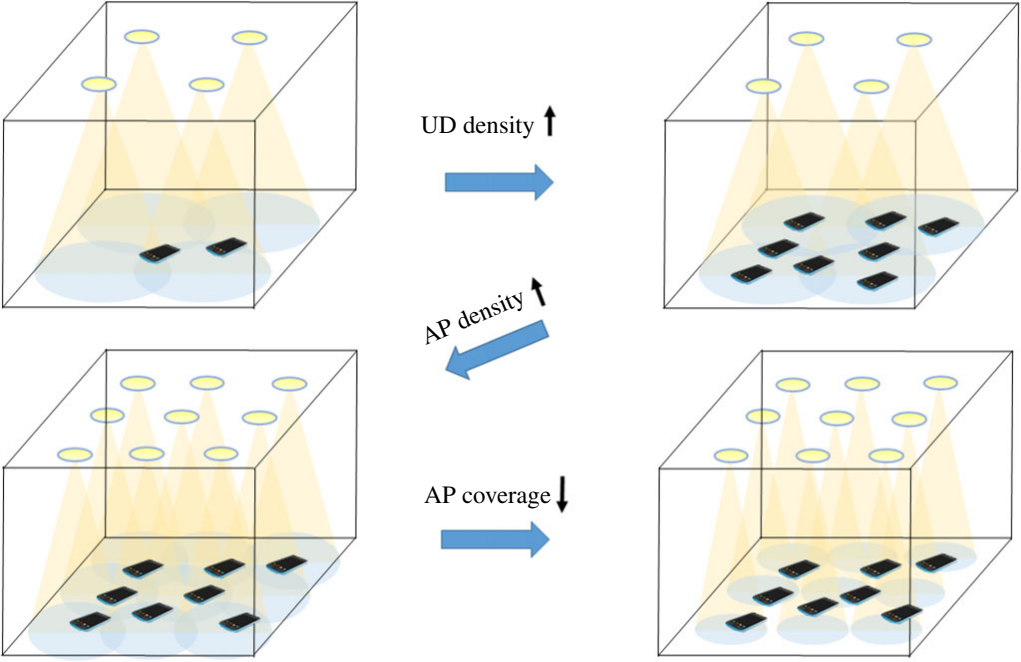
Parameters impacting interference in multiple cell VLC/LiFi systems. (Online version in colour.)

Early research in VLC mainly focuses on the physical layer and single-link implementations to ensure successful point-to-point communication, isolating the transmitter and receiver link into a fixed system. This research improves link performance through signal processing and the introduction of novel modulation techniques. More recent works delve into the system level by deploying cells of multi-point-to-point or multi-point-to-multi-point communication. These multi-AP systems introduce neighbouring cells which naturally induce interference when signals from multiple APs are simultaneously received. Interference can also occur from multiple user transmissions; but little work has analysed interference in the context of OWC uplinks and the impact of in-band uplink on overhead (i.e. AP) signal distribution. This is primarily because many systems incorporate asymmetry by using an alternative medium for uplink. For this reason, our survey focuses on downlink interference, leaving the uplink interference analysis as an open research opportunity. Figure 1 shows the parameters that affect the interference analysis of VLC during densification. These include number of user devices (UDs), AP coverage, AP density and coverage overlap. By increasing AP density, it is believed that the additional capacity provided will deal with the aggregate demands of growing device density. However, higher AP density also implies either smaller AP coverage or increased coverage overlap (and interference). These parameters are also related to transmitter emission pattern including beam width. The emission pattern, AP distribution, device density and the acceptance pattern (device field of view, FOV), determine, and can be used to control, interference in a VLC system.

Wireless capacity can be added to indoor spaces by increasing AP density; but doing so can increase interference. Fortunately, light, with its directional property, offers performance gains with respect to interference. In this paper, we explore the relationship of VLC with radio frequency (RF) models but mainly focus on the unique characteristics of VLC interference mitigation. We discuss recent techniques proposed to improve system performance and describe possible research opportunities.

Note that we will henceforth use the term OWC rather than VLC or LiFi which imply the use of the visible spectrum. Other optical spectra such as ultraviolet (UV) or infrared (IR) are applicable as well as covered by the more general OWC label.

The remainder of the paper is organized as follows: in §2, we overview the unique characteristics of OWC interference. In §3, we introduce interference management techniques for static systems, and in §4 we discuss strategies to dynamically adapt the technique to the characteristics of the environment. Section 5 concludes the paper.

## 2. Interference in optical wireless networks

Interference is a critical parameter when deploying systems where multiple links are simultaneously active and within range of each other. In this section, we identify unique characteristics of the optical medium and OWC network deployments that distinguish OWC interference analysis from the traditional interference analysis techniques used for RF communications.

### (a) Characteristics of optical media

With respect to the physical medium, optical media are characterized in the following ways.

**Figure 2.**
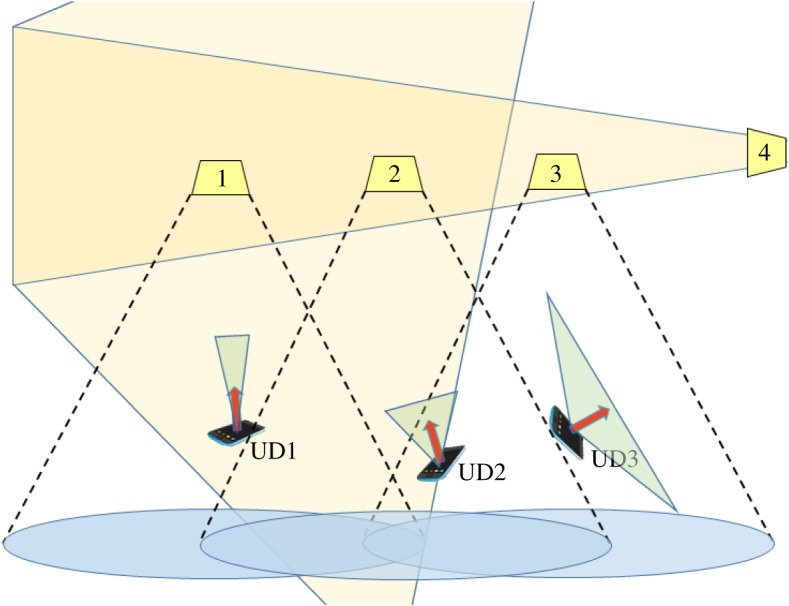
Light-based sources exhibit directionality, divergence and reflections. Receivers and transmitters can have different orientations and fields of view. (Online version in colour.)

— *Directionality of the medium*. The transmitter’s illumination pattern/beam width, as well as the receiver’s FOV, establish the directionality property (a sensor only detects signals in its FOV). Figure 2 illustrates this property through various receivers pointed in different directions, rotations and FOVs with respect to a transmitter. This directionality implies that the relative orientation of the transmitter and receiver has a significant effect on the communication quality [9]. Sub-gigahertz (sub-GHz) RF is modelled as omnidirectional. At higher frequencies, it becomes similar to optical in its directionality.
— *Optical power constraints*. When the optical carrier is also expected to provide illumination, the optical signal must conform to lighting conventions and the nature of human perception including intensity, glare, flicker and colour quality. Also, intensity modulation with direct detection (IM/DD) signals have a different relationship between the constraint (average optical power versus electrical power) and the signal current or voltage. For an electrical signal *X*(*t*), average optical power in IM/DD relates to a constraint on *E*[*X*], whereas average electrical power sets a constraint on the variance of *X*, or *E*[*X*^2^] assuming *X*(*t*) is a 0 mean signal [10].
— *Modulation schemes*. With the use of IM/DD, optical signals transmitted are real-valued and non-negative [11]. This property leads to many modulation variants such as pulse amplitude modulation (PAM), pulse position modulation (PPM) and asymmetrically clipped orthogonal frequency division multiplexing (AC-OFDM) for reconciling light as a carrier.
— *Noise*. Ambient lighting (i.e. sunlight) produces shot noise in OWC systems. DC optical power from ambient light sources can also result in saturation at the receiver, potentially clipping modulated signals intended for reception.
— *Channel characteristics*: Optical signal power consists of line of sight (LOS) and reflected multipath signals (e.g. figure 2, AP number 4). However, most research, with a few exceptions, considers reflections to have negligible impact when an LOS path exists. For example, Komine & Nakagawa [12] show results that conclude that for most environments the total average power in reflections can be ignored.
— *Occlusions*. Light signals are blocked by opaque objects, whereas RF typically can propagate with some degree of attenuation. This can be a security enhancement for limiting eavesdropping on OWC, or it can be a nuisance when moving objects pass between a transmitter and receiver obstructing the LOS path. RF adopts this characteristic in the millimetre wavelengths.

### (b) Interference characteristics unique to optical wireless communications

Interference is widely explored in the RF domain; however, there are differences exhibited due to the properties of the media at different operating points in the electromagnetic spectrum. RF models for interference are insufficient for characterizing light-based models [11,13,14]. In the following, we enumerate some of the important factors to explore beyond the extensive body of work on RF interference.

— *Interference sources*. RF has well-known interference sources such as intra-cell interference, which exists between users that reside within the same cell and inter-cell interference, which is caused by neighbouring cells. In OWC systems, the same types exist except that opaque walls as well as directionality can sequester the inter-cell interference and thus limit its scope (with added benefits of security and privacy). However, OWC has new sources of interference such as uncoordinated sources (conventional lighting) or different OWC technologies (e.g. sensors, cameras or differing OWC products). Related work on this topic includes references [11,15,16].
— *Impact of scale*. Interference in RF systems is invariant to scale. The challenges are very similar across many scales (macro-cells to small cells); however, the containment of LOS light signals means that the physical deployment will impact the nature of interference and will not necessarily be scale-invariant.
— *Gaussian interference model accuracy*. Owing to the directionality of light and receiver FOV, only a subset of the transmitted signal may be received. When only a small number of interfering signals fall within the receiver FOV, the central limit theorem does not hold and interference may not be modelled accurately as a Gaussian distribution since it follows the distribution of the dominant interference source [14].
— *Signal variance*. In IM/DD OWC systems, in contrast to RF ones, the relationship between the optical power constraint and signal variance is modulation-specific. The variance is directly related to the electrical power constraint (i.e. *E*[*X*^2^]) in most RF systems; however, the relationship between the variance and the average optical power constraint (i.e. *E*[*X*]) depends on the modulation used.

### (c) System models for managing interference

**Figure 3.**
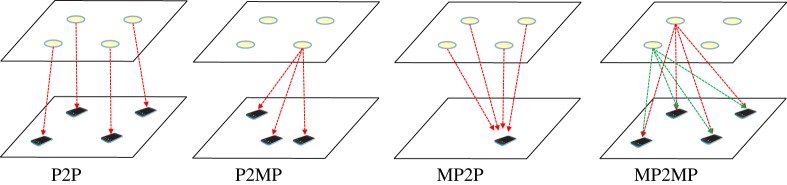
A range of system models for multiple AP-based OWC systems: (1) P2P, (2) P2MP, (3) MP2P, and (4) MP2MP. (Online version in colour.)

A wireless network with multiple simultaneous transmissions can be analysed as a single-coordinated AP or as multiple-coordinated APs. For OWC, we expect that the scope of interfering devices is more constrained due to the directionality of light and the nearness of adjacent APs. This is in contrast to RF-based APs which have much larger spatial scope. Figure 3 shows different system models for downlinks in multiple AP OWC systems. These include (1) point-to-point (P2P), in which each transmitter–receiver pair has a dedicated connection throughout the transmission; (2) point-to-multipoint (P2MP), where an AP can transmit to many devices and maintain several connections according to a specific multiple access technique; (3) multipoint-to-point (MP2P), in which APs synchronize their transmissions to a device, commonly adding overhead but allowing higher user throughput and/or connection reliability; and (4) multipoint-to-multipoint (MP2MP), where devices can receive from several APs while the APs are allowed to transmit to many devices and signal processing is employed to sort out desired signals per user. This is a distinction from single-input–single-output (SISO), single-input–multiple-output (SIMO), multiple-input–single-output (MISO) and multiple-input–multiple-output (MIMO) which focus on the elements from specific devices like sensors, pixels and photodetectors. In this context, a ‘point’ refers to either an AP or a UD.

Each of these models suffers from different types of interference. These include inter-cell interference: UDs share the same resources but are in different cells; intra-cell interference: UDs consume different resources but are within the same cell; cross-cell interference: UDs are in different cells and have different resources (i.e. non-ideal filters within the system or frequency leakage).

Interference and its impact can be characterized by direct and indirect metrics. Table 1 summarizes the critical metrics that are used in the literature to characterize interference in OWC systems. Of course, there are trade-offs among the metrics. Different works attempt to maximize different objectives.

**Table 1.** Summary of performance metrics for evaluating interference.

metric	definition
SINR	signal to interference plus noise ratio, commonly evaluated at the physical layer level to describe the signal quality.
SIR	signal to interference ratio for evaluation of scenarios that are interference dominant.
ASE	area spectral efficiency defined in [17] as the sum of the maximum average data rates per unit bandwidth per unit area.
BER	bit error rate; shows the reliability of the network.
complexity	can represent hardware or software.
robustness	sensitivity to changes in model assumptions or use case.
outage probability	the probability that packets are lost and/or users are not able to access a network or stay connected.
max users	maximum number of users allowed to access the system.
system throughput	aggregate throughput (versus user throughput).
fairness	equal access for all users to the capacity.
EVM	error vector magnitude. An error vector is the difference between the transmitted signal and the received signal. EVM can be defined as the ratio between the error vector mean and the original signal’s mean.

Of this list, we identify a subset as best for establishing and benchmarking the performance of multiple AP systems. These are (1) system throughput, (2) sustained link speed of a single user, and (3) system complexity. However, in practice, evaluating published work on multiple access OWC systems is challenging due to disparate assumptions and operating conditions. To help with this challenge, we break down the techniques into categories to help permit fair comparison. These are described next.

## 3. Interference management techniques

Up to this point, we have discussed the challenges associated with OWC APs including the nature of the optical physical channel models. In this section, we survey and classify existing techniques intended to manage interference in the context of multiple access in OWC systems. We begin with a summary of nomenclature and classification and then relate key results from the literature onto this classification.

**Figure 4.**
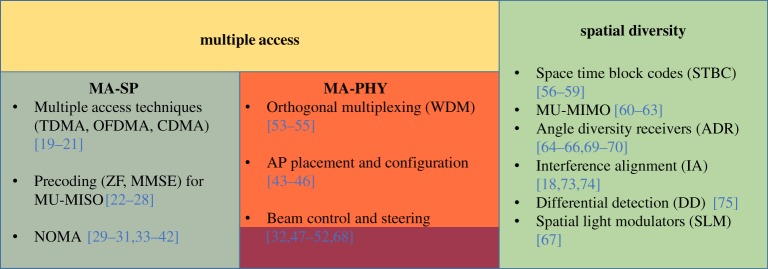
Categorization of interference management. (Online version in colour.)

A review of the state-of-the-art related to OWC interference studies yields a range of terms similarly applied. These terms include the following: *rejection* is the strictest term used to describe completely removing interference from a system. It is normally employed in physically isolated techniques that can effectively separate resources or channels. *Coordination* is done in systems that attempt to either use interfering signals to their advantage or arrange transmissions in a way to cause the least possible interference. Coordination is normally done in networked or synchronized systems. *Interference alignment* is a scheme developed for RF [18] that increases the degrees of freedom (d.f.) of the interference channel by the alignment of signal spaces (in time, frequency, space and codes) such that signals arrive relative to the receiver to yield interference-free or near-interference-free reception. This technique is performed in systems that can achieve diversity in the signal space. *Avoidance, mitigation, cancellation and suppression* are ways to minimize interference. Although cancellation sounds as strong as rejection, most of the literature uses it to describe minimizing interference where some interference effect remains. These techniques are adopted in systems with fixed resources that deploy resource allocation or load balancing. *Management* is a term that is usually used broadly to describe each of the previous systems that aim to combat interference effects in any manner. Using the term ‘interference management’ to cover the range of nomenclature, we classify techniques in OWC into two main categories (figure 4). These are:

— *Multiple access* (*MA*). This class consists of the techniques that allow an AP or set of APs to distribute defined resources across a set of users. It is divided into two further categories:
  (i) *MA-signal processing* (*MA*-*SP*). This class of techniques applies pre-processing at a transmitter to enable distinction of signals at the receiver, such as employing orthogonal multiple access. The received signal is the superposition of the intended signal and some number of interfering signals.
  (ii) *MA-physical isolation* (*MA*-*PHY*). This class of techniques isolates signals by using the physical characteristics of the channel such as using orthogonality of physical resources (e.g. wavelength division multiplexing) or by leveraging the properties that are distinct to OWC, such as directionality or receiver FOV.
— *Spatial diversity*. This class encompasses both of the PHY and SP cases and is concerned with receiving data by many spatially unique channels or multiple PDs then performing signal processing to have them de-correlated.

Next we consider the state of the art for interference management in each of these classes and corresponding to the different system models (figure 3). For each class, we distill specific and narrow results from representative papers.

### (a) Multiple access-signal processing

**Figure 5.**
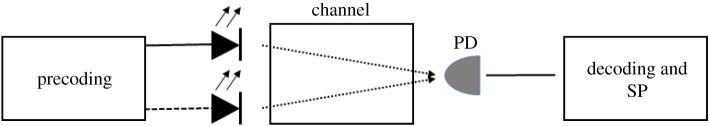
MA-SP configuration.

In the first instance (signal processing, figure 5), we have three sub-cases of (i) multiple access, (ii) precoding for multiuser-MISO (MU-MISO) and (iii) non-orthogonal multiple access (NOMA). These are described below.

#### (i) Multiple access techniques

Multiple access refers to how a set of resources can be distributed to serve multiple UDs. The characteristics of the medium used are reflected in the techniques applied towards sharing the channel. In the basic case, resource distribution provides fairness from a single AP to multiple users. The other case involves a receiver detecting multiple sources. Here the resolution can include more complex coordination among APs or more advanced coding techniques.

Marsh *et al.* [19] provide an early study, assuming IR channels, of the effects of the classical fixed (static) channel reuse techniques including time-division multiple access (TDMA), frequency-division multiple access (FDMA) and code division multiple access (CDMA), comparing them with cell radii as an important parameter. Via simulation, the authors conclude that in a small cell (3 m) and the use of optical orthogonal codes (OOCs), performance is dominated by co-channel interference with an irreducible BER performance for smaller cells (less than 1.5 m). The exception is the use of CDMA with m-sequences.

Kim *et al.* [20] study frequency carrier allocation in VLC systems. Channel performance is analysed through EVM, and they perform experiments to confirm their results for mitigating inter-cell interference. These works [19,20] focus on static channel reuse which helps in increasing SINR values and decreasing error vectors but unfortunately degrades overall spectral efficiency and subsequent system throughput.

For the visible spectrum, Jung *et al.* [21] analyse reducing inter-cell interference in multi-cellular VLC cells by using OFDMA (multiple access) cell partitioning. This work seeks to improve spectral efficiency using a reduction in the guard band in the frequency partitioning by using a filter bank-based multi-carrier (FBMC) to suppress the effect of OFDM sub-carrier sidelobes. The work reports improvements of 1.5× capacity and spectral efficiency compared to OFDMA.

#### (ii) Precoding schemes for multiuser-multiple-input-single-output

The techniques mentioned here employ *cooperative transmission or joint transmission* (JT) which allows transmission from multiple transmitters to a single VLC receiver, similar to MP2P systems as shown in figure 3. MU-MISO combines multiple distributed transmitters to act as a single cell or AP.

An approach using synchronized time of arrival (TOA) from multiple sources was introduced by Prince *et al.* [22]. This method was designed to source identical data to improve signal strength at a targeted receiver in the lighting field. The work shows that cooperative transmissions reduce inter-symbol interference (ISI); however, the system adds a significant overhead and delays transmissions affecting delay-intolerant systems drastically, and it would not scale with many or highly mobile users.

Chen *et al.* [23] adapt JT to the downlink transmission in an optical atto-cell network to mitigate co-channel interference (CCI) improving overall cell-edge user throughput and SINR. The system assumes communication between the LEDs and the UDs to establish who the best transmitter is, calculate SINR based on the highest signal received and then the devices form a look-up table based on the possible transmitters. This introduces overhead to the system specially for mobile users. The JT scheme employs frequency allocation which inherently means reduced spectral efficiency and throughput but the authors show an improvement over static frequency reuse cases.

Park *et al.* [24] study an interference mitigation scheme for VLC systems deployed in aircraft employing a minimum mean square error (MMSE) and zero forcing (ZF) algorithm for cancelling interference signals. Then successive interference cancellation (SIC) is used with optimal ordering. The performance of their scheme is evaluated using BER, comparing ZF, ZF with SIC, MMSE and MMSE with SIC, with the latter giving the best (lowest) BER performance. However, no quantification of latency introduced by the technique is reported.

Yu *et al.* [25] tackle MU-MISO in indoor broadcast VLC systems by applying two precoding techniques, linear ZF and ZF-dirty-paper-coding (ZF-DPC) to eliminate interference between users under the illumination constraints with ZF-DPC outperforming ZF specifically when two users are close to each other. However, the authors assume perfect channel state information (CSI) which is not practical. Ma *et al.* study robust MMSE linear precoding for MU-MISO VLC broadcast systems assuming imperfect CSI to create a more robust precoder in [26]. This system requires a powerline backbone communication systems controller to allow data sharing and synchronization for successful communications. Pham *et al.* [27] explore ZF to suppress multiple user interference and they propose a generalized inverse-based ZF scheme to maximize the system sum rate, which they report to outperform pseudo-inverse design through numerical simulations.

Li *et al.* [28] design two transceivers in an indoor MU-MISO VLC setting, an optimal one based on a linear MMSE formulation and a simplified transceiver based on ZF precoding and notice performance in different user densities. They also report results for mean square error versus number of users.

MU-MISO precoding can be very beneficial for users to help increase their data rates as well as the system throughput. However, there is much complexity on the transmit side that needs to be clarified. The transmitters are considered connected, synchronized and/or have channel information regarding the receivers in most works mentioned above. Some works assume transmit precoding as well. The backhaul network must be able to handle such overhead, and as more devices enter the system, the technique does not scale gracefully. While these systems appear promising, there needs to be more investigation to establish scaling limits with respect to number engaged transmitters UDs.

#### (iii) Non-orthogonal multiple access

NOMA permits the full use of the channel bandwidth by any transmitter, relying on superposition coding at the source and successive interference cancellation at each receiver. Many works have been published on the topic of NOMA in 5G networks [29,30].

Marshoud *et al.* [31] propose a gain-ratio power-allocation (GRPA) scheme that factors each user’s channel conditions in a NOMA downlink (DL) VLC system. This requires a central controller. They show enhancement in system performance when compared to static power allocation, they also study the effect of LED transmission angles, employing a technique similar to reference [32], as well as receiver FOVs because they can enhance the channel gains. However, the work does not consider receiver misalignment which can be introduced with device mobility.

Kizilirmak *et al.* [33] compare the performance of NOMA in a DL VLC system to OFDMA while considering the impact of cancellation error in SIC receivers. Yin *et al.* [34] study NOMA SIC error impact in a LiFi system. Meanwhile, in another work, Yin *et al.* [35] derive coverage probability and ergodic sum rate in DL VLC NOMA for two scenarios: (1) achieving a guaranteed quality of service and (2) an opportunistic best-effort service. They compare NOMA to TDMA and show results relating to the LED transmission semi-angle. Then in [36] they extend their work to provide a theoretical framework that analyses the performance of VLC NOMA and characterizes its performance gains over orthogonal multiple access (OMA). Marshoud *et al.* [37] analyse BER performance in DL NOMA VLC for both perfect and imperfect CSI.

There are works that opt for solutions other than SIC such as Guan *et al.* [38], who study joint detection (JD) of VLC signals employing NOMA for the uplink (UL) where they suggest that transmitters use JD which is based on the maximum-likelihood detection and provides enhanced BER results compared to SIC. They maximize the minimum distance between constellations to improve performance by pre-distorting the phase of the CSI. They confirm their work experimentally using feedback channels from the receivers. Wang *et al.* [39] use joint detection and decoding in their energy efficient transceiver design for DL VLC NOMA systems where they propose a power allocation scheme based on finite alphabet inputs. Chen *et al.* [40] study SIC-free NOMA in DL VLC systems and use constellation partitioning coding and uneven constellation de-mapping.

A low complexity power control allocation that aims at fairness under optical intensity constraints by maximizing the sum log user rate is studied by Yang *et al.* [41]. Chen *et al.* [42] study efficient and low complexity power allocation in MIMO NOMA VLC and propose a normalized gain difference power allocation.

While most works mention that NOMA is attractive for OWC systems, especially indoors, because the channel at a fixed location/orientation is nearly deterministic and the SNR is relatively high (in the absence of blockage), there is still a need for channel estimation as performance is impacted by shadowing, receiver mobility and orientation. NOMA has the advantage of allowing full resource usage and therefore best spectral efficiency. The main drawbacks of this approach are complexity, error propagation, and latency added due to the use of SIC. There is room for new solutions that find a trade-off between SIC and joint detection in terms of complexity while keeping a tolerable delay.

### (b) Multiple access-physical isolation

**Figure 6.**
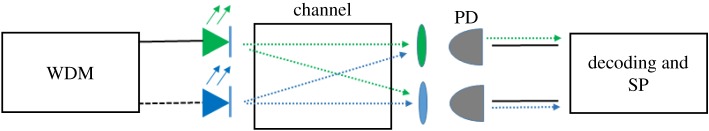
Typical MA-PHY model with two optical wavelengths. (Online version in colour.)

Here, we consider the implications of the physical layer characteristics on the nature of interference. This class includes sub-cases of (i) AP positioning, (ii) beam control and (iii) orthogonal multiplexing (figure 6). Each is reviewed in turn.

#### (i) Access point placement and configuration

**Figure 7.**
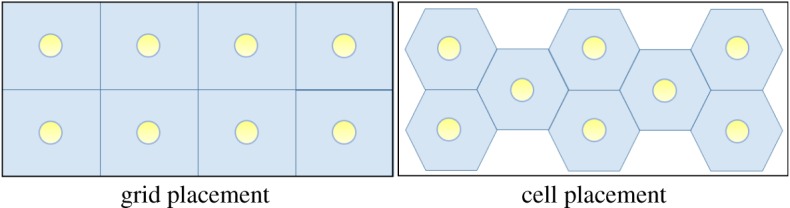
Lighting-based APs are usually grid-like. But cellular arrangement can be used effectively. (Online version in colour.)

The conventional model for an RF AP is a single omni-directional unit spanning multiple rooms in the service to a locale (e.g. an apartment). Coverage of a larger facility (building or campus) is realized by replication of APs. OWC APs, being primarily LOS-based, can service much smaller zones such as individual rooms, or can be replicated in larger rooms (e.g. open office seating). Clearly, there are important design considerations for the height, spacing and beam width of the OWC APs especially if they are also intended to provide lighting (figure 7). Current work on AP placement and configuration explores this system design problem.

Wang *et al.* [43] investigate LED placement to reduce SNR fluctuation for a multi-user VLC setting in an attempt to maintain consistent user performance at all locations within a room. The authors study the performance of 12 LED lamps placed in a circle at the centre of a room with additional LED sources at the edges. The results show small SNR variability in comparison to the more common LED placements in the centre of the room. They also study how this placement increases ISI near the edges and propose a remedy using zero-forcing at the receivers. Stefan *et al.* [44] focus on maximizing a room’s ASE through optimal placement of APs. Their results show that for a receiver with wide FOV the transmitters are better placed far from each other while at lower FOVs a receiver experiences less interference. The optimal configuration is found between these bounds. Their results also show that ASE is reduced when lighting constraints are imposed in the optimization. Shashikant *et al.* [45] propose different arrangements for placing the VLC APs and compare their coverage aspects. They define simple metrics, namely coverage, interference fraction and interference to coverage, to establish which arrangement is better. Results show that a hexagonal set-up for VLC APs gives best coverage and least interference fraction. Vegni *et al.* [46] study optimal LED placement in indoor VLC networks under constraints of illumination and data rate outage. They propose two techniques—attocell minimization (ACM), aims to minimize the number of attocells that can serve a given number of users (to minimize unnecessary interference) and attocell user maximization (AUM), which maximizes the number of users that an attocell can serve. The results show that a large half-power semiangle and lower data rate per user requirement can realize an improved guaranteed performance. The authors did not study changing parameters on the receive side such as orientation or FOV which can highly affect their results.

AP placement is an interesting topic with more to investigate, and in particular, the impacts of mobility and device density. The introduction of steerable APs also adds a new dimension to improving system performance.

#### (ii) Beam control and steering

The aforementioned cases consider static lighting and AP scenarios in which the source intensity parameters are provisioned for a particular operating point. With the use of beam control including beam width (transmitter FOV) and beam steering (directionality), new optimizations result as well as options to support adaptive performance to varying device location and data traffic.

Rahaim *et al.* [32] study SINR and VLC cell zooming while maintaining constant illumination in indoor VLC networks. The concept here is to adapt RF cell zooming but for OWC while operating within lighting constraints. The technique allows a dynamic change of cell size, which can be implemented through cell power reduction or by physically changing the emission pattern of the transmitter to allow for better communication for the users. Results indicate comparisons to frequency reuse scenarios and discuss combination scenarios of LOS versus multipath regions. The technique can also be very useful in minimizing overhead from handover if cells can grow to cover users in larger footprints. This system can also be very interesting if studied dynamically for mobile users with randomly oriented devices within a room to show more practical results.

Valagiannopoulos *et al.* [47] suggest a transmitter with a nanoslit metasurface that increases the directivity of the transmitted beams and therefore improves the received SIR. Here the nanoslits act as very directed transmitters with tiny cone beams that are able to concentrate the emitted power into small beams thereby suppressing interference between LEDs. The authors argue that while their system is susceptible to receiver rotations and misalignment, the angles and sizes of the receivers and the nanoslits demonstrate that these perturbations do not affect performance. Additional work will be required to establish viability under practical operating conditions.

Beam steering can also be used at the transmit side, either as a narrow or diffuse beam [48–50]. By using a narrow beam, interference can be mitigated by source control delivering signal exactly to a receiver. With the use of high-speed control (mirrors or other spatial light modulators), rapid steering between receivers is possible with rates in the order of kilohertz for point-to-point cycles. O’Brien [49] has successfully demonstrated such a system using IR signals. Once using a laser diode source, data rate can be very high relative to LED sources. However, this type of system is not appropriate for lighting and must factor eye safety requirements in its design.

Another MA-PHY technique is proposed by Chang *et al.* [51] involving a low-cost spectrum sensor array at the receiver intended to reject interference on incoming wavelength division multiplexing (WDM) signals. The receiver is also designed to cancel out ambient light interference. The technique is based on a filtering optimization formulation leading to optimal weights that allow SIR to be maximized. This approach requires timing synchronization between the employed sensors to work successfully.

In addition to controlling the directionality of a source, the FOV and pointing angle of a receiver can be controlled to improve interference rejection. Abdalla *et al.* [52] propose a dynamic FOV receiver intended to isolate a channel through dynamic control of receiver FOV. Applications include responding to changes in device orientation, position and velocity, and in supporting AP selection when traversing a larger set of OWC APs. A demonstration system supports predicted performance improvements in realized SNR.

#### (iii) Orthogonal multiplexing

This technique facilitates co-location of multiple non-interfering (orthogonal) signals that can be decoded selectively by independent receivers. (Note that the classifications of multiple access and multiplexing converge when considering only two endpoints, e.g. time division duplexing (TDD).)

Liu *et al.* [53] demonstrate a bi-directional LED VLC system using TDD protocol aimed at mitigating reflection interference. Their system has LEDs for both uplink and downlink and involves mirrors placed to investigate the behaviour of reflections. This method addresses interference but at the cost of delays due to cycling the channel among participating devices. Wu *et al.* [54] consider specific modulation schemes for adjacent cells including on–off keying (OOK), pulse width modulation (PWM) and PPM. Experiments indicate error-free transmission at 1 Mb s^−1^ and 6.25 Mb s^−1^. Butala *et al.* [55] propose a multi-wavelength VLC system design, including an analysis of the relationships among the source channel wavelengths, relative intensities and optical filtering to realize maximum SNR while minimizing cross-talk at the receiver.

Exploiting WDM in OWC systems is an attractive means to gain capacity. However, it needs careful design to meet the relevant illumination requirements. These include (1) light distribution and intensity levels including minimizing glare, each dependent on the lighting use case; (2) providing satisfactory spectral power distributions for colour rendering, essential for humans; and (3) avoiding visible temporal discontinuities in colour or spatial distribution of light. Each of these is surmountable, but requires consideration when designing the modulation approach involving WDM or combinations of WDM with time division multiplexing (TDM).

### (c) Spatial diversity

**Figure 8.**
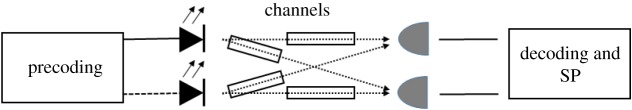
Typical diversity model.

Diversity techniques exploit the ability to simultaneously source multiple signals that can be combined at a receiver, the ability to receive sources from multiple detectors at a receiver, or some combination of the two techniques. The diversity class of interference management includes sub-cases of (i) space time block coding, (ii) multiuser-MIMO, (iii) angular diversity, (iv) interference alignment, (v) differential detection, and (vi) spatial light modulators (figure 8).

#### (i) Space–time block coding

Space–time block coding (STBC), first introduced for RF communications, has been established as a method to allow diversity in a system to achieve very low BER while saving power [56]. Ntogari *et al.* [57] analyse the performance of STBC techniques in indoor diffuse optical wireless systems. The authors use discrete multi-tone modulation (DMT) to mitigate the effect of ISI caused by a channel impulse response and compare the performance of two Alamouti STBC schemes to SISO and maximum ratio combining (MRC) schemes. Their results show that STBC schemes can increase system coverage and capacity and decrease the transmitted optical power required. Biagi *et al.* [58] propose an MIMO-PPM-STBC in a VLC set-up under the constraint of trace-orthogonal matrices focusing on achieving middle ground between high transmission rate and low BER. This performance contrasts with systems that only focus on one of the two sacrificing the other. The authors compare their system performance to other work (e.g. [57]) and show that they achieve middle performance trading off BER and transmission rate.

Meanwhile Shi *et al.* [59] construct an experiment to test the performance of MISO VLC networks using STBC where they employ two red, green and blue (RGB) LEDs as transmitters and a single receiver to test STBC-OFDM coding. They investigate performance in the overlapping region of the two transmitters. They report a total throughput of 500 Mb s^−1^ adding that the free space transmission range can be extended to 5 m. BER is reported at less than 10^−5^. However, their system does not account for any mobility.

While STBC can be beneficial in achieving low BER, there is a relatively high complexity involved with this approach. This includes the complexity associated with achieving syncronization of distributed transmitters.

#### (ii) Multiuser multiple-input-multiple-output

Hong *et al.* [60] investigate the performance of block diagonalization (BD) precoding to eliminate multiuser interference, reducing the terminal (receiver) complexity level in a multiuser MIMO (MU-MIMO) setting. They test the performance on different receiver FOVs as well. Their simulation results show SNR up to 40 dB. The system is able to theoretically achieve 100 Mb s^−1^ at a BER of 10^−6^ while 70° and 50° are considered for receiver FOV.

Pham *et al.* [61] also employ BD. In this case for precoding of broadcast channels to find a lower bound for the sum-rate maximization of all users in an indoor space. Their results show the impact of photodiode (PD) rotations and user locations on interference. Wang *et al.* [62] investigate MU-MIMO OFDM for VLC systems. In this work, they propose to evaluate a precoding matrix based on MMSE or ZF for each OFDM subcarrier with the goal of eliminating multiple user interference. Their results indicate that MMSE outperforms ZF when the optical power is low.

Lian *et al.* [63] study an indoor MU-MIMO VLC system using spatial multiplexing with a centralized power allocation and with four different decentralized transmitted power allocation algorithms. Each employs multiple LEDs and PDs, the latter having different orientation angles for better SINR. CDMA is used to accommodate the users, and receiver time–space MMSE filters are used to reduce the multiple access interference. They consider parameters such as shadowing, dimming control, illumination level and transmitted power quantization. They mention that the distributed techniques show an overall lower computational complexity. In terms of BER two of the distributed algorithms, weighted decentralized multi-detector power allocation joint optimization (PAJO) and partial decentralized multi-detector PAJO, outperform all other proposed methods. While using their centralized scheme helps in shadowing scenarios in getting twice the data rate compared to not knowing the shadowing information.

MIMO schemes support increases in system capacity and related performance but at a the cost of complexity. Some of the systems mentioned above employ precoding which helps in relieving interference but assumes transmitter connectivity and the availability of CSI which is not always the case nor is it necessarily accurate. In support of this scheme, indoor VLC channels tend to be more deterministic but only in a static study. When device mobility is introduced, CSI changes and any static precoding matrices quickly become obsolete.

#### (iii) Angle diversity receivers

**Figure 9.**
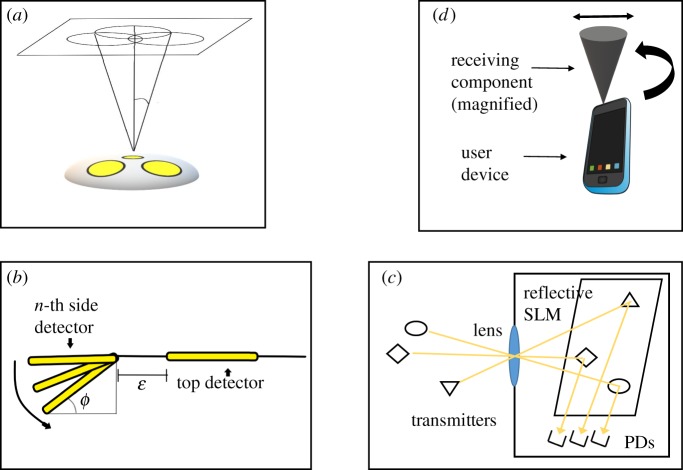
Proposed receiver designs: (*a*) 3 PD ADR Receiver, adapted from [65], (*b*) generalized ADR, adapted from [66], (*c*) reflective MEMS SLM optical receiver [67], (*d*) D-FOV tracking receiver [68]. (Online version in colour.)

Angle diversity receivers (ADRs) rely on the ability to discern the angle of arrival of an incident transmitted signal [64]. They are typically designed as a hemisphere with multiple PDs arranged on the surface (figure 9*a*,*b*) but other designs using masks suspended over array receivers can be used [69].

Most works on this topic focus on using ADR receivers in MIMO settings and for optical channel decorrelation. ADRs can also be used to mitigate inter-cell interference (ICI). Haas *et al.* [70] explore using an ADR receiver and signal combining schemes (from the many photodiodes on the receiver) to increase system SINR and achieve higher area spectral efficiency. The authors report that the ADR employing maximum ratio combining gives the best performance and provides 40 dB in SINR improvement over a single PD setting. Their results show that the ADR outperforms single PD receivers in both SINR and ASE.

Chen *et al.* [65] propose a signal combining method named optimum combining (OPC) which incorporates interference correlation and studies non-LOS (NLOS) reflections. An interesting discussion in this work relates to NLOS reflections and their impact on SINR. The results indicate that the first-order reflections degrade performance only slightly because the first-order reflections bounce off the walls and fewer users are near the walls of a room. However, the second-order reflections emanating from the ceiling impact SINR negatively and cannot be completely eliminated through manipulation of FOV. To combat this effect from reflections, they propose using ADRs and compare a single PD receiver to ADRs with multiple elements (three and seven elements; figure 9*a*). Their results show that increasing the number of PDs (increasing diversity) improves the ability to reduce interference caused by reflections. And that the increased number of PDs degrades performance when reflections are not present due to the limited FOV of the PDs, all while assuming a fixed effective area.

Chen *et al.* [66] study SINR variability in indoor multi-cell VLC systems by proposing an optimized angle diversity receiver. This follows related work in SNR variability [43] (§3bi). The authors propose a generalized ADR structure comprised a top detector with optimizable side detectors that can change their angles of inclination, *ϕ* (figure 9*b*). Results show promise in the ability to minimize SINR fluctuation through optimizing parameters such as number of detectors, inclination angle and the combining scheme for the signals received by the PDs. However from their description of their ADR, they do not optimize *ϵ*, which they define as the gap between the top detector and the side one although that area where *ϵ* presides is very useful and important for communication quality, they also do not study NLOS reflections. Most importantly, they do not study receiver orientation effects and so the proposed optimal inclination angle they reach through simulation is only optimized for a horizontally fixed receiver.

Each of the works indicate that improvements in SINR can be achieved at a receiver by employing more PDs. However, they do not account for the variations in receiver orientation, which is critical to link performance [9,71,72].

#### (iv) Blind interference alignment

Less stringent in its requirements, blind interference alignment (BIA) [73] is a variation on IA that does not require CSI and specifically the channel coefficient values at the transmitters. BIA only needs the knowledge of the channel coherence times (CSI is assumed to be known at the receiver). A benefit of BIA in VLC is that the transmitting LEDs do not need to cooperate. Wu *et al.* [74] analyse BIA for MU-MISO indoor VLC systems proposing a filter-pair-based scheme in which the receiver has a single PD and multiple receive filters, instead of reconfigurable antennas, which control the receive mode. They show that their system outperforms OMA schemes in terms of spectral efficiency and degrees of freedom.

#### (v) Differential detection

Ryoo *et al.* [75] propose a differential optical detection system that engages both the transmit and receive elements. The transmitter consists of an LED and a polarizer; the receiver comprises two PDs each with a polarizer to provide differential detection. Then the transmitted polarized signal is received by one PD and blocked out of the other so that in the differential part of the system everything else cancels out except the desired signal and some noise. Experimental results verify their system for one or two interference sources but their EVM worsens when more interference sources are introduced but BER is still in the range of 10^−3^.

Limitations of this approach exist when scaling to many UDs or in the presence of occlusions or variations in device orientation which may mask only a single channel. The use of a polarizer inherently limits incident signal strength as well.

#### (vi) Spatial light modulators

Spatial light modulators (SLMs) can be used reflectively or transmissively to manipulate or modulate optical signals. One approach leverages an array of mirrors to focus and enhance signal strength directed towards one or more PDs. The array of mirrors can be individually steered to reflect light from the target. All or a subset of the mirrors. By pointing the mirrors the signal strength of the target is increased (maximized) and the noise from other sources is minimized. Bare PDs do not have this feature. Chau *et al.* [67] propose the use of SLM for MIMO VLC receivers as it has the ability to dynamically control the optical channel and to support its decorrelation. The design of the receiver is based on using an imaging receiver, a lens and a reflective SLM at the image plane that directs the incoming light signal in the direction of an array of photodetectors (figure 9*c*). This method has been successfully demonstrated. It shows promise in being able to isolate the channels at the receiver, support diversity combining, and is relatively integratable into a working system.

## 4. Managing system dynamics

The techniques discussed up to this point represent the building blocks for the construction of high-performance OWC systems. The introduction of device mobility, orientation, density and traffic use priorities each introduce new complexities for maintaining consistent performance. These factors motivate addressing the development of adaptive approaches that can react to changes in system state. The main topics considered here are (a) resource allocation and load balancing, (b) coordinated transmission, (c) combined beamforming, and (d) beam and FOV control.

### (a) Resource allocation and load balancing

Many works in the literature aim to optimize resource allocation and load balancing with different end goals in mind. We focus on the works that target interference mitigation.

Mondal *et al.* [76] propose a coordinator to estimate the interference level (IL) of users which the authors evaluate based on the SINR of the user and their distance from the AP. The coordinator then assigns a visible light multicolour logical channel based on the IL metric. They reserve part of the channel set for control and hand-off purposes. Some priority is given to the lowest level interferers in getting the least interfering channel, as they form a queuing model and show results in case of low traffic, high traffic and prioritized traffic dropping probabilities as well as SINR cumulative distribution function (CDF). However, the results are not compared to a baseline scheme for a generalization of the system performance.

Li *et al.* [77] investigate different VLC cell formations with different frequency reuse (FR) patterns such as (1) unity frequency reuse (UFR) which uses up all the resources and suffers from interference in cell edges. (2) Non-unity FR, where the reuse factor=2 in a hybrid VLC/wireless fidelity (WiFi) scenario. They then show the performance of different cell formations as (1) combined transmission where 2 VLC APs join to transmit the same data to the user, giving better SINR but less bandwidth efficiency which drives them to propose (2) vectored transmission (VT) where ZF is employed to serve many users in the interference region simultaneously, if cells are merged as well then it becomes JT with VT, example VT-16 is 16 cells merged together. They study centralized and distributed approaches for a proportionality fairness scheduler focusing on throughout, fairness and area spectral density. The proposed VT-16 shows the most promising results but the most overload on the system to use all 16 LEDs for a single user and so no interference would be experienced since the system has 16 APs total. The system is static and is not considering user mobility as well as device orientation. To accommodate system dynamics, Li *et al.* [78] build a user-centric system, as opposed to a network-centric system, that employs VT to mitigate ICI. Their results show that VT-user centric clusters outperform network centric ones in average user throughput bearing in mind the presence of impending issues that need to be accurate/solved for the system to perform well such as user position estimation and possible system blockages.

To improve load balancing results, Soltani *et al.* [79] consider two metrics, signal strength (SS) and AP traffic, in making an AP selection in LiFi cellular networks while assuming random receiver orientation, however, it is assumed that the receiver angles are uniformly distributed along their dynamic ranges which is not practical in a real life setting and draws attention to a much needed accurate statistical modelling of UD orientation angles. They also study LOS and NLOS cases in their SINR study. The authors propose a central controller to carry out the load balancing and discuss their results in terms of average user throughput, satisfaction level and fairness index showing an edge to their proposed metric in comparison to only SS technique.

Wang *et al.* [80] study load balancing under device orientation and shadowing in indoor hybrid LiFi/RF networks. Their results show better performance than other load balancing schemes, such as joint optimization algorithm, threshold-based access algorithm and random access algorithm, in terms of user quality of service level while attaining lower complexity as well. Hammouda *et al.* [81] perform a two-step resource allocation process to allocate both zone and user level resources evaluating ASE and fairness. They study their system performance in a room-scale transmission scenario using the simulation tool CandLES [82]. However, they assume fixed receiver orientation. Zhang *et al.* [83] propose a predictive system that exploits location and delay information to achieve a better delay-throughput trade-off, through anticipating user association in an indoor VLC network. They assume perfect knowledge of the users’ locations as well as *a priori* knowledge of the users’ wireless traffic distribution and then form an optimization problem that maximizes the sum rate for the duration of several future time slots weighted by the evolving queue backlog of each user over many future time slots. They compare their anticipatory association (AA) with responsive association (RA), which maximizes the sum rate at a current time slot weighted by the current queue backlog of the user. They report that their system outperforms RA achieving better trade-off between the average system queue backlog and the average per user throughput. They also note that their study indicates that the overall system average delay can be reduced when AA is employed. However, their choice of mobility model was the random waypoint model which is not practical and the assumption of perfect receiver location is not quite robust. Designing a predictive system is a step in the right direction but much more analysis and study is needed to delve deeper in the variations of this dynamic system and its reliability. There is definitely room for innovation in this area.

### (b) Coordinated transmission

Coordinated transmission involves multiple transmitters working together to produce signals decoded by one or more receivers. This includes (i) coordinated and non-cooperative cases and (ii) coordinated and cooperative cases.

#### (i) Coordinated non-cooperative transmission

Self-organizing interference coordination in optical wireless networks is the topic of the work of Ghimire *et al.* [84]. The authors investigate interference coordination in an aircraft cabin scenario with an OFDMA/TDD system. The authors investigate a busy burst (BB) scheme in which APs are required to share common channels. APs intending to reuse a resource need to listen in on BB messages without centralized controllers aiding them and evaluate the CCI they would cause to a user and decide based on that whether to transmit or wait. They add a heuristic that stops a user from reserving the resource after the user has had a fair share of resources. The authors compare their approach to static resource partitioning and reveal improvement in fairness and spectral efficiency. However, it would be useful to see how many users are allowed in the system, how much delay is caused due to contention and the overall system dropping probability.

**Figure 10.**
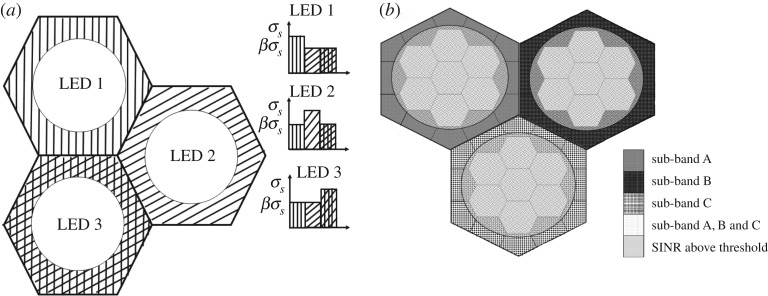
Cell planning. (*a*) Colour cell planning [86] and (*b*) FFR [85].

Chen *et al.* [85] study fractional frequency reuse (FFR) which they consider a compromise between full frequency reuse and static partitioning schemes. In FFR, the entire bandwidth is partitioned into several sub-bands depending on the reuse factor which they choose to be 3. The cell is divided into an interior region and an exterior region based on an SINR threshold. If an SINR is above the threshold then the device is considered to be in the interior and may use the full frequency band otherwise the device is in the exterior region and can use a sub-band (figure 10*b*). Owing to this partitioning they provide a power control factor *β* to help edge users get higher power than centre ones. This method improves the cell-edge user and overall system throughput.

Zhou *et al.* [86] analyse a multicolour VLC system adopting soft frequency reuse (SFR) based interference coordination but performed on colour planning. They divide the VLC attocell hexagon into an inner circle which uses two colours then the outer region would use the third colour of the RGB division making sure the neighbouring cell gets a different cell edge colour (figure 10*a*). While accommodating illumination requirements, they propose a static scheduler that adjusts power control to give centre cell users the minimum required communication performance to mitigate interference for cell edge users, as well as a dynamic scheduler for dense scenarios when the static scheduler is not sufficient to combat interference for the cell edge users. The dynamic scheduler limits colour usage in some attocells according to interference intensity and under SINR demands of all the UDs. It is intended to function in one of two modes: (1) a distributed stage followed by a centralized stage jointly to adjust colour usage, or (2) a greedy approach based on dividing the hexagon cell into rings which would then get colours according to their location. They compare their results with CD which is the one colour per cell plan and NoICIC which is the plan where a cell is allowed to use all colours. They show results based on inter-LED distances but the system has a very high overhead, requires two scheduling phases which can provide delays for highly mobile users, and also depends on reliable feedback channels which may not be practical.

Of the works that study interference coordination employing NOMA (§3a(iii)), Kashef *et al.* [87] study an interference coordination scheme in that entails two transmission schemes to maximize a network utility function; one which uses orthogonal multiple access and the other uses NOMA. A central controller decides which scheme to use based on user location and other system parameters. Another example is by Zhang *et al.* [88], who explore grouping users based on their locations to reduce interference then they optimize power allocation for NOMA taking residual SIC interference into account.

#### (ii) Coordinated and cooperative transmission

Bai *et al.* [89] propose a coordinated transmission scheme based on a bipartite graph formed in the downlink. They form a max–min problem to maximize the minimum user rate and perform an allocation scheme derived from the optimization problem. They divide the possible scenarios into two cases (1) when a device is in an overlapping region between two cells and so the cells would allocate the same sub-carriers, labelled ‘coordinated transmission’, and (2) when the device is not in an overlapping region and thus the same sub-carrier is not assigned as in an overlapping region. This case is labelled ‘interference mitigation’. They use their graph theoretic algorithm backward forward marking (BFM) to do the allocation. They then compare their throughput and fairness results to round robin and proportional fairness schemes and show that BFM gives better performance. They consider first-order reflections in their work but make the assumption of perfect CSI at both transmitter and receiver which is not a robust assumption.

These areas are very interesting; there is a need to identify ways to realize maximum performance obtainable from the diverse set of physical layer techniques under dynamic conditions. We would hope that future work would define upper bounds on number of supported users on repeatable benchmark traffic and mobility configurations. We note that some of the aforementioned techniques will be challenging to scale to many APs (e.g. the use of synchronized transmitters).

### (c) Combined beamforming

**Figure 11.**
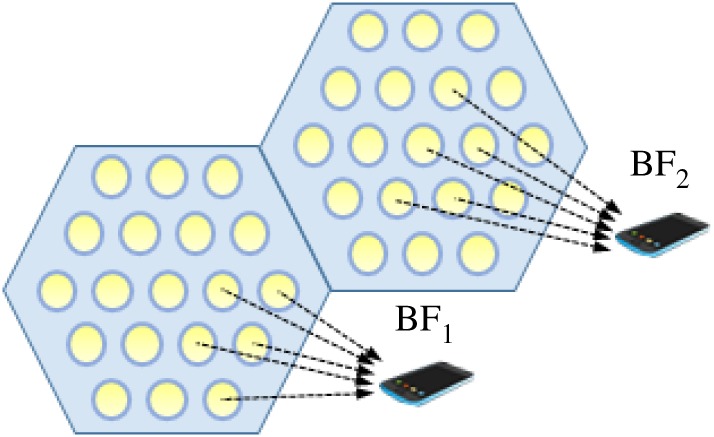
Combined beamforming scenario. (Online version in colour.)

Combined beamforming (figure 11) employs cells comprised multiple LEDs. Within each cell the individual LEDs are modulated to deliver one signal to a UD depending on their location.

Mostafa *et al*. [90] suggest that since JT required too much overhead and high synchronization between transmitters, coordinated beamforming (CB), adopted from RF, would allow for less transmitter collaboration for interference mitigation. This concept is studied in a downlink multi-cell MU-MISO VLC system. They assume that an attocell consists of a single transmitter with multiple LEDs and can serve a number of users each having a receiver with a single PD and each user is served by a single attocell. Taking intra-attocell and inter-attocell interference as well as illumination requirements and receiver noise into consideration, they form an optimization problem to find the best linear MMSE beamforming design. The authors employ linear beamforming design in a perfect CSI set-up and a more robust imperfect CSI setting. While their CB scheme exploits the spatial domain for multiplexing and interference mitigation, the results show that in comparison to JT, CB is sometimes close to performance of JT when there are many transmitters but for specific user distributions it is very limited in comparison to JT. The closer users are together, the harder it is to beamform messages intended to specific users specially when they are served by only a single attocell. The authors provide a possible solution to add coordinated scheduling to alleviate this problem for certain user distributions by serving them in different time slots in order to achieve resource partitioning. Based on the results in the paper, solving the high-density case needs more study and analysis.

### (d) Beam and field of view control

Beam and FOV control can also be used to address dynamic user behaviour. Examples include aforementioned work [52] and references [32,48,49] which can be considered dynamic as they adapt to system changes to fulfill the user requirements. In [52], the receiver adapts to changes in the user location, orientation and velocity by evaluating the best (transmitter-FOV) pair per receiver dynamic change to allow it to receive the best SNR. While reference [68] (figure 9*d*) is more active in adapting the FOV and orientation to allow the user the best SNR quality and is able to change them dynamically with system changes. By contrast, work that proposes dynamic beam and steerability adapt the transmit side to change along with the user density, location and overall motion within the room to allow for better coverage and communication quality. Both areas are very interesting and have room for more analysis in the system scale.

### (e) Combinations

Here, we discuss systems that generally combine some of the building blocks discussed in §3. Cui *et al.* [91] propose jointly employing a colour and code strategy to mitigate interference in indoor VLC femtocells. First they use WDM between different VLC cells from the colours red, green and blue, then they assign phase-shifted maximum length pseudo noise (PN) sequences to LEDs that have the same wavelength. They also use orthogonal Walsh–Hadamard (WH) code for different users within a cell to combat intra-cell interference. According to their design they have no intra-cell interference but inter-cell interference is limiting the system. They evaluate their system performance in relation to parameters such as closest LED distance, user density in a cell and the dimming level of the LED. However, their system complexity is relatively high and requires accurate synchronization.

Adnan-Qidan *et al.* [92] study user-centric BIA design in a VLC system to relieve the transmitters from complexity of designing a precoding matrix in which they employ a reconfigurable PD in an ADR configuration (§3c(iii)). They propose two schemes depending on how the user clusters are connected. One scheme considers a broadcast channel for each cluster named KM-sBIA and the other scheme, KMtopBIA, allows each cluster to divide into graphs that depend on the users’ connectivity. They report that their schemes outperform ZF (transmit precoding method) in both network and user centric settings in BER and user rate.

## 5. Conclusion

OWC promises to provide a huge boost to the capacity of indoor AP networks including those coupled to the lighting function as VLC. However, the properties of the optical spectrum and the anticipated increase in density of mobile-UDs requires a revisit to interference management for this media type. In this paper, we explore the state of the art with respect to multiple user optical access and develop a classification of current technical approaches for exploiting this technology and managing interference. There are a few key takeaways from the survey which we summarize here:

(i) VLC provides links that have unique properties of light leading to methods and performance unique to OWC.
(ii) When we adopt and replicate VLC for APs, we need to address interference among UDs and among the multiple APs.
(iii) Many interesting techniques exist (spatial multiplexing, WDM, ADR, etc.) each with different considerations for interference.
(iv) Techniques can be combined and optimized for static scenarios, but ultimately we need to study how they interact and can adapt to follow the dynamics introduced by device mobility, orientation and data traffic models.
(v) Future work will address system dynamics including optimization and management in the context of overall network performance adaptation.

Finally, we emphasize the importance of being able to compare the strengths of future innovations by ensuring that a common set of operating conditions are adopted. With such a foundation we would expect the most critical performance metrics will be (1) system throughput, (2) sustained single-user link speed, and (3) system complexity. We hope to evaluate future work based on these primary metrics.

## Data accessibility

This article has no additional data.
